# Supplementary material for: Dermoscopic image segmentation based on Pyramid Residual Attention Module
Source: PLoS One. 2022 Sep 16;17(9):e0267380. doi: 10.1371/journal.pone.0267380 (PMC9481037; doi:10.1371/journal.pone.0267380)
Supplement: Attachment — Submitted filename: Answers for Reviewer 2.docx [file pone.0267380.s001.docx]

[Answers for Reviewer 2’ Comments]

First of all, we would lie to thank you for all that you commented for our paper.

Point 1: Figure 2 and Figure 3 are so messy that the reader cannot understand the architecture of the proposed module. Please use a more concise figure to illustrate the PRA network.

Response 1:

We are grateful for this suggestion.

In this regard, we change the figure 2 to this appearance. And the figure 3 is our designed to present the process of PRA from accepting the feature to get the output. The detail is narrated at the 3.2.

We propose a streamlined and effective synthesis module called the pyramidal residual attention (PRA) module. To better understand the principle of the module's composition, we use a simple convolutional neural network model of feature learning concept to illustrate. Fig3 shows the flow of the PRA module for processing feature maps. Taking the deep level of the network as an example, the size of the input image and the output feature map is 16×20. Firstly, the features are processed on the basis of the original map using 32 convolution kernels size of 3×3 as a result of this level of information extraction. At the same time, the input feature maps are compressed by down-sampling once, using a 2×2 pooling layer with a step size of 2, concentrating the semantic information and ignoring the useless spatial information. The output is fed into a feature pyramid to learn the feature information got from different sizes of receptive fields. The feature sets of multiple receivers are used as the input for the channel attention. Channel attention takes the results of different convolutional blocks to derive the information weight of each channel through a pooling layer. Thus these options can enhance the input to different degrees to focus the attention of the model. Finally, after recovered the size by the deconvolution layer, the feature maps connect with the output of the residual block to obtain the output of the module.

Point 2: There are four identical convolution modules above each feature pyramid, and I hope the authors explain what they do.

Response 2:

We are grateful for this suggestion.

The four convolution is our feature pyramid. It is used to study the different knowledge from different convolution. Because convolution has their own perceptual fields. The details like following:

Feature pyramids are feature extractors that incorporate different kinds of convolutional kernels. Because the convolution operation is pixel-by-pixel, features of different sizes can be learned efficiently by these convolution kernels. Four convolutional kernels of 226 1×1, 3×3, 5×5 and 7×7 are used to process the input in parallel. Multiple different convolutional kernels are able to learn different knowledge from different perceptual fields [45], adding more semantic and spatial information to the results of the original 229 single 3×3 convolutional kernel. The stride of the convolution kernel is set to 1, which 230 does not change the size of the image while feature extraction, so that the features of the image can be substantially preserved and provide information security for subsequent learning.

Point 3: There are many errors in the paper, please check the paper in detail by the authors. For example, there are missing spaces at the end of the sentences in lines 217 and 241.

Response 3:

We are grateful for this suggestion.

In this regard, we carefully checked and corrected the mistakes.

Point 4: All experiments in this paper were done on the ISIC dataset. Please conduct the same experiments on other datasets to verify the generalization ability of the proposed model. The explanation for the experiment is inadequate. More analysis should be added to demonstrate the core idea of the whole paper.

Response 4:

We are grateful for this suggestion.

In this regard, we add the generative experiment of KvasirSEG datatset to valid our model. The detail is following.

| Network | year | DC | MIOU | SP | ACC | SE | Para(M) |
| --- | --- | --- | --- | --- | --- | --- | --- |
| U-Net[8] | 2015 | 89.40±**5.33** | 82.12±**6.64** | 94.01±3.48 | 94.66±3.47 | 95.94±3.46 | 31 |
| AttU-Net[9] | 2018 | 89.16±5.71 | 81.81±8.30 | 93.75±3.45 | 94.40±3.44 | 95.08±3.44 | 33.3 |
| DenseASPP[53] | 2018 | 89.63±6.64 | 82.76±9.33 | 94.25±3.84 | 94.91±3.84 | 95.59±3.85 | 33.8 |
| CE-Net[53] | 2019 | 89.95±5.72 | 83.07±8.32 | 94.35±**3.24** | 95.01±**3.21** | 95.69±**3.20** | 24 |
| ADAM[12] | 2019 | 88.22 | 81.37 | **96.68** | **98.28** | 91.04 | - |
| CA-Net[11] | 2020 | 88.55±6.19 | 80.99±8.65 | 93.57±3.77 | 94.22±3.76 | 94.89±3.76 | 2.7 |
| PRAU(proposed) | 2021 | **90.88**±5.39 | **84.41**±8.01 | 94.73±3.46 | 95.40±3.43 | **96.09**±3.41 | 32 |

We use the KvasirSEG dataset to evaluate our model. in the above experimental setting, we initialize the images of different sizes to 384×384 size. the KvasirSEG dataset consists of 1000 images, we randomly disorganize them and take 500 as the training set, 100 as the validation set, and finally 400 as the test set. Using the same data enhancement method as the above dataset, the effect is shown in Fig. The specific metrics are shown in Table. It is shown in the experiments that the addition of attention affects the segmentation performance of the model, and in some cases, it even increases the learning burden of the model significantly. For example, CANet, after using attention instead, makes the model not as effective as it should be. Combining PRA with UNet and using the PRA module to replace the bottom module in UNet can improve the model performance. The improvement of the segmentation effect of UPRA can be clearly felt from the figure, which is significantly more than several other models.

Point 5: Some missing key references about segmentation, and residual attention [1,2,3,4] as these methods have been widely used in these works.

Response 5:

We are grateful for this suggestion.

In this regard, we The following papers are cited as a supplement.

1. Lu et al.[26] proposed a CO-attention mechanism which can enhance the model to capture remote information.
2. Lu et al.[40] proposed the Shrinkage loss function to balance number of data of the different classes.

Thanks for your comments.
