# Supplementary material for: Dermoscopic image segmentation based on Pyramid Residual Attention Module
Source: PLoS One. 2022 Sep 16;17(9):e0267380. doi: 10.1371/journal.pone.0267380 (PMC9481037; doi:10.1371/journal.pone.0267380)
Supplement: Attachment — Submitted filename: The Answers for Reviewer 1.docx [file pone.0267380.s002.docx]

[Answers for Reviewer 1’ Comments]

First of all, we would lie to thank you for all that you commented for our paper.

Point 1: The authors have done a good job in addressing my comments. There are still some grammar mistakes, and the authors should further improve their writing, especially the abstract.

Response 1:

These are improving of our papers, as follow:

For the first, the abstract:

The diversity and complexity of skin lesion images are the challenges of dermoscopic image segmentation. Convolutional neural networks(CNNs) have played an essential role in addressing it in recent years. In this paper, to segment this kind of image better, a novel feature extraction module PRA(Pyramid Residual Attention) is designed from a new perspective. PRA consists of a residual module, an attention module, and a pyramid module. We use the denoising function of the encoder-decoder structure and combine it with a multi-scale feature pyramid. The channel attention is to monitor the feature extraction process of the pyramid to ensure the efficiency of the PRA. The PRA Network is composed of shallow, middle, and deep layers. Both the shallows and middle layer consist of two PRA modules, and the deep layer consists of the single one. The shallow, medium, deep layers in PRA Network link by down-sampling and up-sampling to improve the segmentation effect. The network is subjected to ablation experiments on the ISIC 2018 dataset to verify the effectiveness of the PRA module and achieve better results in all metrics. Our method achieved better results for Dice coefficient (DC) indices equal to 93.37% and 89.47%, for MIOU indices up to 87.94% and 82.14% respectively for the segmentation tasks on the ISIC 2018 dataset and ISIC 2017 dataset. The PRA Network is more effective compared with existing advanced methods.

We correct the mistakes and trim the word surplus in abstract.

The second, details:

1、The dermoscopy is designed to provide the physician with high-resolution images of abnormal parts of the patient’s epidermis.

Change to

The dermoscopy provides the physician with high-resolution images of abnormal parts of the patient's epidermis.

……

We have used the grammarly to check mistake of all sentences, and correct them one by one. But many use of passive voice and objective voice are confused. We try our best to improve our writing and become better.

We are grateful for this suggestion. In this regard, we carefully checked and corrected the mistakes.

Thanks for your comments.
