# Supplementary material for: Dermoscopic image segmentation based on Pyramid Residual Attention Module
Source: PLoS One. 2022 Sep 16;17(9):e0267380. doi: 10.1371/journal.pone.0267380 (PMC9481037; doi:10.1371/journal.pone.0267380)
Supplement: Attachment — Submitted filename: answers for editors.docx [file pone.0267380.s003.docx]

[Answers for academic editor’ Comments]

Comments ：Please improve the language as the reviewer suggested.

Response 1:

We try our best to improve our paper, and using the grammarly to guarantee the grammar correct. In order to make the paper normal, we motivate the good paper’s rhetoric. Thanks for your comprehension and supporting.

There are changes in our paper compared to the last version.

Firstly, the abstract is changed from

The diversity and complexity of skin lesion images are the challenges of dermoscopic image segmentation. Convolutional neural networks(CNNs) have played an essential role in addressing it in recent years. In this paper, to segment this kind of image better, a novel feature extraction module PRA(Pyramid Residual Attention) is designed from a new perspective. PRA consists of a residual module, an attention module, and a pyramid module. We use the denoising function of the encoder-decoder structure and combine it with a multi-scale feature pyramid. The channel attention is to monitor the feature extraction process of the pyramid to ensure the efficiency of the PRA. The PRA Network is composed of shallow, middle, and deep layers. Both the shallows and middle layer consist of two PRA modules, and the deep layer consists of the single one. The shallow, medium, deep layers in PRA Network link by down-sampling and up-sampling to improve the segmentation effect. The network is subjected to ablation experiments on the ISIC 2018 dataset to verify the effectiveness of the PRA module and achieve better results in all metrics. Our method achieved better results for Dice coefficient (DC) indices equal to 93.37% and 89.47%, for MIOU indices up to 87.94% and 82.14% respectively for the segmentation tasks on the ISIC 2018 dataset and ISIC 2017 dataset. The PRA Network is more effective compared with existing advanced methods.

to

We propose a stacked convolutional neural network incorporating a novel and efficient pyramid residual attention module (PRA) for the task of automatic segmentation of dermoscopic images. Precise segmentation is a significant and challenging step for computer-aided diagnosis technology in skin lesion diagnosis and treatment. The proposed PRA has the following characteristics: Firstly, we concentrate on three widely used modules in the PRA. The purpose of the pyramid structure is to extract the feature information of the lesion area at different scales, the residual means is aimed to ensure the efficiency of model training, and the attention mechanism is used to screen effective features maps. Thanks to the PRA, our network can still obtain precise boundary information that distinguishes healthy skin from diseased areas for the blurred lesion areas. Secondly, The proposed PRA can increase the segmentation ability of a single module for lesion regions through efficient stacking. Finally, we incorporate the idea of encoder-decoder into the architecture of the overall network. Compared with the traditional networks, we divide the segmentation procedure into three levels and make the pyramid residual attention network (PRAN). The shallow layer mainly processes spatial information, the middle layer refines both spatial and semantic information, and the deep layer intensively learns semantic information. The basic module of PRAN is PRA, which is enough to ensure the efficiency of the three-layer architecture network. We extensively evaluate our method on ISIC2017 and ISIC2018 datasets. The experimental results demonstrate that PRAN can obtain segmentation performance comparable to state-of-the-art deep learning models under the same experiment environment conditions.

Secondly, the capitalization and voice problems of the first letter of the sentence are all solved, because these correcting are so many, they will be highlighted in the track version.

Thanks for your comments.
